# Polygenic risk score, psychosocial environment and the risk of attention-deficit/hyperactivity disorder

**DOI:** 10.1038/s41398-020-01019-6

**Published:** 2020-10-02

**Authors:** Søren D. Østergaard, Betina B. Trabjerg, Thomas D. Als, Clara Albiñana Climent, Florian Privé, Bjarni Jóhann Vilhjálmsson, Marie Bækvad-Hansen, Jonas Bybjerg-Grauholm, David M. Hougaard, Merete Nordentoft, Thomas Werge, Ditte Demontis, Preben B. Mortensen, Anders D. Børglum, Ole Mors, Esben Agerbo

**Affiliations:** 1 Department of Affective Disorders, Aarhus University Hospital - Psychiatry, Aarhus, Denmark; 2 Department of Clinical Medicine, Aarhus University, Aarhus, Denmark; 3 The Lundbeck Foundation Initiative for Integrative Psychiatric Research (iPSYCH), Aarhus, Denmark; 4 NCRR - National Centre for Register-based Research, Aarhus University, Aarhus, Denmark; 5 Department of Biomedicine and Center for Integrative Sequencing, iSEQ, Aarhus University, Aarhus, Denmark; 6 Department for Congenital Disorders, Statens Serum Institut, Copenhagen, Denmark; 7 Copenhagen Research Center for Mental Health - CORE, Mental Health Center Copenhagen, Copenhagen University Hospital, Copenhagen, Denmark; 8 Research Institute of Biological Psychiatry, Mental Health Center Sanct Hans, Copenhagen University Hospital, Roskilde, Denmark; 9 Center for Genomics and Personalized Medicine, Aarhus, Denmark; 10 CIRRAU - Centre for Integrated Register-based Research at Aarhus University, Aarhus, Denmark; 11 Psychosis Research Unit, Aarhus University Hospital – Psychiatry, Aarhus, Denmark

**Keywords:** Psychiatric disorders, Medical genetics, ADHD

## Abstract

The objective of the present study was to investigate whether the polygenic liability for attention-deficit/hyperactivity disorder (ADHD) and the psychosocial environment impact the risk of ADHD in interaction or independently of each other. We conducted a register- and biobank-based cohort study of 13,725 individuals with ADHD and 20,147 randomly drawn population-based controls. These 33,872 cohort members were genotyped on the Infinium PsychChip v1.0 array (Illumina). Subsequently, we calculated the polygenic risk score (PRS) for ADHD and extracted register data regarding the following risk factors pertaining to the psychosocial environment for each cohort member at the time of birth: maternal/paternal history of mental disorders, maternal/paternal education, maternal/paternal work status, and maternal/paternal income. We used logistic regression analyses to assess the main effects of the PRS for ADHD and the psychosocial environment on the risk of ADHD. Subsequently, we evaluated whether the effect of the PRS and the psychosocial environment act independently or in interaction upon the risk of ADHD. We found that ADHD was strongly associated with the PRS (odds ratio: 6.03, 95%CI: 4.74–7.70 for highest vs. lowest 2% liability). All risk factors pertaining to the psychosocial environment were associated with an increased risk of ADHD. These associations were only slightly attenuated after mutual adjustments. We found no statistically significant interaction between the polygenic liability and the psychosocial environment upon the risk of ADHD. In conclusion, we found main effects of both polygenic liability and risk factors pertaining to the psychosocial environment on the risk of ADHD—in the expected direction.

## Introduction

Attention-deficit/hyperactivity disorder (ADHD) is a childhood-onset neurodevelopmental disorder with a prevalence of approximately 5–7% among children/adolescents^1,2^. ADHD is characterized by hyperactivity, inattention, and impulsiveness^3^—all to an extent, which causes significant burden on those affected, their families and society as a whole^4–10^.

The specific causes of ADHD remain largely unknown, but a number of twin studies have suggested that the disorder is among the most heritable (heritability recently estimated at 74% based on data from 37 twin studies^11^ in the entire spectrum of mental disorders^11–13^. Accordingly, recent studies focusing on both common- and rare genetic variants have identified genes associated with ADHD^14–16^. Also, a number of early adverse psychosocial factors have consistently been linked to ADHD^17–21^. Furthermore, there is a growing body of literature supporting the hypothesis that genetic and psychosocial risk factors do not only have direct effects on the risk of ADHD, but also interact^22–24^. However, most studies on the combined effect of genetic and psychosocial risk factors for ADHD are based on relatively small samples of patients and healthy controls volunteering for study participation, who have self-reported historical data on their psychosocial environment^25–29^. The combination of the use of healthy controls and self-reported historical data on psychosocial environment introduces a substantial risk of selection and recall bias^30,31^. Relatedly, most prior gene–environment interaction studies in ADHD have focused on candidate genes in the dopaminergic and serotonergic system that were not replicated in the recent GWAS of ADHD^15,25–29^. Therefore, studies of the effect of interaction between genetic risk and psychosocial environment upon the risk of ADHD, which avoid the abovementioned threats to internal validity are needed. Here, we aimed to conduct such a study using data from the Lundbeck Foundation Initiative for Integrative Psychiatric Research (iPSYCH) case-cohort^32^. Specifically, the aim of the study was twofold:

I. *To investigate the main effects of the polygenic liability for ADHD and the psychosocial environment on the risk of ADHD*: Here, we will investigate the association between a polygenic risk score (PRS) for ADHD, maternal/paternal education, maternal/paternal work status, maternal/paternal income and maternal/paternal history of mental disorder and the risk of developing ADHD in a population-based sample.
II. *To investigate polygenic by psychosocial risk factor interaction on the risk of ADHD:* Here, we will test whether there are interactions between the genetic liability (PRS) and the risk factors pertaining to the psychosocial environment (maternal/paternal education, maternal/paternal work status, maternal/paternal income and maternal/paternal history of mental disorder) with regard to the risk of developing ADHD

## Methods

### Setting

This study was based on data from the iPSYCH case-cohort, which consists of 86,189 singletons born in Denmark between May 1, 1981 and December 31, 2005, who resided in Denmark on their 1st birthday^32^. The case-cohort comprises 57,377 individuals registered with either schizophrenia, bipolar disorder, unipolar depression, autism or ADHD diagnosis in the Danish Psychiatric Central Research Register^33,34^ as well as 30,000 randomly drawn population controls^32^. These individuals were genotyped using the Infinium PsychChip v1.0 array (Illumina) and psychosocial information for each individual (parental education, work status, income, and psychiatric history) was extracted from nationwide Danish registers. Since the establishment of the iPSYCH case-cohort was not based on opt-in participation^32^, the risk of selection and attrition bias is minimal. Furthermore, as proxy information on the psychosocial environment is registered routinely for all individuals with an address in Denmark^17^, there is virtually no recall bias either. Therefore, this dataset allows us to investigate the combined contribution of genetic and psychosocial risk factors for ADHD in a population-based sample virtually without the impact of the typical biases affecting studies of this type.

### Population

In this study, we focused on the individuals that were included in the iPSYCH case-cohort due to an ADHD diagnosis (ICD-10 code F90.0) as well as the iPSYCH population controls for whom there were valid genotypes and who had parents that were both born in Denmark^32^. To further mitigate confounding by population stratification, we computed the orthogonalized Gnanadesikan-Kettenrin robust Mahalanobis distance of the ten leading ancestral principal components, and excluded individuals who had a logarithm-distance larger than three (1120 with ADHD and 1730 population controls)^35,36^. This led to inclusion of a total of 33,872 individuals, namely 13,725 with ADHD and 20,147 population controls. There were 771 sibling constellations (2–4 cohort members with the same parents) within this population. For 454 (58.9%) of these constellations, all siblings were in the ADHD group, for 154 (20.0%), all siblings were among the population controls, and for 163 (21.1%) the sibling constellation represented a mix of individuals with ADHD and population controls. A total of 750 (97.3%) of the sibling constellations consisted of two siblings. The median age at diagnosis of ADHD was eleven years (interquartile range: 9 years) and total of 593 (4.3%) of the individuals with ADHD were also registered with a diagnosis of oppositional defiant disorder or conduct disorder (as defined by Wimberley et al.^37^) either prior to or at the time of the ADHD diagnosis.

### Data sources

For the 33,872 individuals in the study cohort, we extracted data from six sources, namely the Danish Civil Registration System (link between children and parents—as well as vital status)^38^, the Danish Psychiatric Central Research Register (diagnoses for all individuals assessed or treated for mental disorders at a psychiatric hospital in Denmark)^33,34^, the Danish Education Register (education)^39^, the Danish Register on Personal Labour Market Affiliation (work status)^40^, the Danish Register on Personal Income and Transfer Payments (income)^41^, and the Danish Neonatal Screening Biobank^42^ (genotypes generated via the Infinium PsychChip v1.0 array (Illumina) for obtaining PRS for ADHD)^32^. Linkage of information from these six sources is made possible by the unique personal registration number, which is assigned to all individuals residing in Denmark^38^.

### Definition of genetic variable (PRS for ADHD)

The PRS for ADHD for each individual was estimated as described in Demontis et al^15^. The 23 genotyping batches of the iPSYCH sample were split into five groups of approximately equal size, aiming for an equal number of ADHD cases within each group. Five leave-one-out analyses were then conducted, using four of five groups as discovery datasets for estimation of SNP effect sizes, while estimating PRS for the remaining target group. The GWAS meta-analyses of the discovery samples were conducted using an SNP list filtered for minor allele frequency >0.01 and an imputation threshold score above 0.8 intersecting across batches. INDELs and variants in the extended MHC region (chromosome 6: 25–34 Mb) were also removed. Meta-analysis and “LD-clumping” of significant SNPs were conducted using the ricopili pipeline^43^. PRS was then estimated for each target sample using a range of meta-analysis *p*-value thresholds (5 × 10^−8^, 1 × 10^−6^, 1 × 10^−4^, 1 × 10^−3^, 0.01, 0.05, 0.1, 0.2, 0.5, 1.0), multiplying the natural log of the odds ratio of each variant by the allele-dosage of each variant. Whole-genome PRS were obtained by summing values over variants for each individual. The PRS was standardized within target groups. The significance of the case-control score difference was tested by standard logistic regression including principal component and dummy variables indicating genotyping batch as covariates and the proportion of variance explained was estimated for each *p*-value threshold by comparing the full model with a reduced model without PRS and thus including covariates only (i.e. Nagelkerke’s *R*^2^). The standardized score for the *p*-value threshold with the highest Nagelkerke’s *R*^2^ (*p* < 0.2) was used in the subsequent analyses. The standardized PRS for ADHD was divided into 50 groups each representing 2% of the distribution among the randomly drawn population controls.

### Definition of variables pertaining to the psychosocial environment

While we have previously used a register-based operationalization of Rutter’s indicators of adversity in relation to ADHD^17^, this was not possible in the present study due to restrictions enforced by the Danish Neonatal Screening Biobank. Therefore, in this study, we focused on the psychosocial environment in the family of the cohort members instead, as there is a well-known association between this construct and concrete adverse incidents among children^44–50^. Specifically, we focused on maternal/paternal history of mental disorders, maternal/paternal education, maternal/paternal work status, and maternal/paternal income.

#### Maternal/paternal history of mental disorders

At the date of birth of the cohort members, we assessed whether their parents were registered with a diagnosis of a mental disorder in the Danish Psychiatric Central Research Register^33^. The following categories were defined based on diagnoses from the International Classification of Diseases, 8th revision (ICD-8), which was used as diagnostic reference from 1969 to 1993 and from the International Classification of Diseases, 10th revision (ICD-10), which was used from 1994 and onwards: Schizophrenia and related disorders (ICD-8: 295.x9, 296.89, 297.x9, 298.29–298.99, 299.04, 299.05, 299.09, 301.83. ICD-10: F20-F29), Mood disorders (ICD-8: 296.x9 (excl. 296.89), 298.09, 298.19, 300.49, 301.19. ICD-10: F30-F39), Neurotic, stress-related, and somatoform disorders (ICD-8: 300.x9 (excl. 300.49), 305.x9 305.68, 307.99. ICD-10: F40-F48), and other mental disorders not listed under the categories above (ICD-8: 290–315. ICD-10: F00-F99)^51^. The categories were hierarchical and mutually exclusive such that schizophrenia and related disorders trumped mood disorders, which trumped neurotic, stress-related, and somatoform disorders, which trumped other mental disorders.

#### Maternal/paternal education

Information regarding the parents’ highest completed level of education in the year of the birth of the cohort members was extracted from the Danish Education Register^39^ using the following levels: primary school, high school or vocational education (e.g. carpenter or bricklayer), short- or medium-length higher education (e.g. nurse, schoolteacher and bachelor level university degrees), long academic education (master level university degrees or PhD).

#### Maternal/paternal work status

Information regarding the parents’ primary work status in the year prior to the birth of the cohort member was extracted from the Integrated Database for Labor Market Research^40^ using the following levels: unemployed or otherwise outside the labor marked, student in education, blue collar worker, self-employed, clerical worker or leading wage-earner.

#### Maternal/paternal income

The parents’ income in the year prior to the cohort members’ birth was extracted from the Registers on Personal Income and Transfer Payments^41^. The income was scaled to 2004 level using the price index from the World Bank. The income of the mothers and fathers of the randomly drawn population controls was used to create income quintiles. Individuals with missing values were placed in the lowest quintile.

### Statistics

The associations between the PRS for ADHD, the risk factors related to the psychosocial environment and ADHD were assessed using logistic regression. Odds ratios and 95% likelihood ratio-based confidence intervals were computed. For the main effects of the PRS for ADHD and the risk factors related to the psychosocial environment, crude odds ratios for ADHD adjusted for sex and year of birth were computed. To assess whether the impact of the psychosocial factors was mediated through the genetic liability for ADHD, we calculated odds ratios that were also adjusted for the PRS. Furthermore, to investigate whether the PRS effect was partly explained or confounded by the psychosocial environment, we calculated odds ratios that were fully adjusted for all psychosocial factors. To explore the polygenic liability distribution across case-control status and the factors pertaining to the psychosocial environment, we calculated population marginal means^52^. Lastly, we evaluated whether the PRS (continuous) interacted with the risk factors pertaining to the psychosocial environment upon the risk of ADHD. As a sensitivity analysis, we challenged the necessity for excluding ancestral principal components outliers, by repeating the analyses outlined above, without exclusion of individuals (1120 with ADHD and 1730 population controls) based on ancestral principal components outliers^53,54^. Analyses were conducted using SAS 9.4 and R version 3.5.1.

### Ethics

The study was approved by the Danish Health Data Authority, the Danish data protection agency, The Danish Neonatal Screening Biobank Steering Committee and the Danish Scientific Ethics Committee.

## Results

**Fig. 1:**
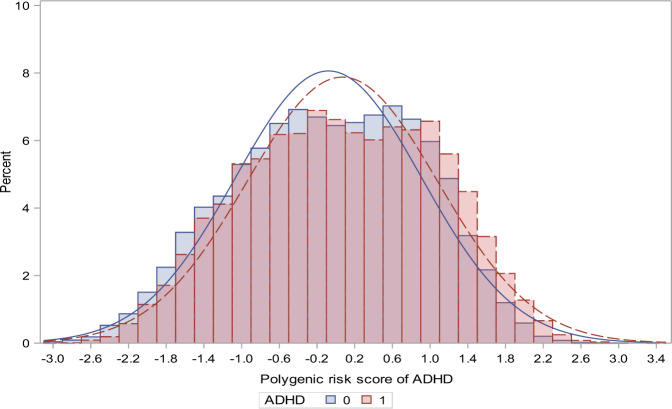
ADHD Polygenic Risk Score Distributions. Comparison between individuals with ADHD and uniform randomly population-based individuals.

The characteristics of the cohort members—including information regarding the PRS for ADHD and the risk factors related to the psychosocial environment—are shown in Table 1. The distribution of the PRS for ADHD for the cohort members with ADHD and the randomly selected population controls is shown in Fig. 1.

**Table 1:** Distribution of polygenic risk score, parental history of mental disorders and socioeconomic factors for the ADHD cases and the randomly drawn population controls.

		ADHD cases		Controls	
		*N*	(%)	*N*	(%)
	**Sex**				
	Male	10,120	(73.73)	10,203	(50.64)
	Female	3605	(26.27)	9944	(49.36)
	**Polygenic risk score**^a^				
	50	513	(3.74)	339	(1.68)
	40	267	(1.95)	384	(1.91)
	30	240	(1.75)	406	(2.02)
	20	248	(1.81)	427	(2.12)
	10	274	(2.00)	400	(1.99)
	1	154	(1.12)	433	(2.15)
Mother	**History of mental disorder**^b^				
	Schizophrenia and related disorders	69	(0.50)	50	(0.25)
	Mood disorders	105	(0.77)	88	(0.44)
	Neurotic, stress-related, and somatoform disorders	309	(2.25)	204	(1.01)
	Other Psychiatric disorder	276	(2.01)	183	(0.91)
	No psychiatric disorder	12,966	(94.47)	19,622	(97.39)
	**Highest obtained education**				
	Primary school^c^	6413	(46.72)	5983	(29.70)
	High school or vocational education	5368	(39.11)	8830	(43.83)
	Short- or medium-cycle higher education	1736	(12.65)	4437	(22.02)
	Long-cycle higher education or PhD	208	(1.52)	897	(4.45)
	**Working status**				
	Unemployed or otherwise outside the labor marked	3851	(28.06)	3506	(17.40)
	Student in education	485	(3.53)	505	(2.51)
	Blue collar worker	5421	(39.50)	6464	(32.08)
	Self-employed	174	(1.27)	335	(1.66)
	Clerical worker or Leading wage-earner	3794	(27.64)	9337	(46.34)
	**Income**				
	Lowest quintile	3741	(27.26)	4017	(19.94)
	Second quintile	3143	(22.90)	4024	(19.97)
	Third quintile	2735	(19.93)	4030	(20.00)
	Fourth quintile	2362	(17.21)	4032	(20.01)
	Highest quintile	1744	(12.71)	4044	(20.07)
Father	**History of mental disorder**^b^				
	Schizophrenia and related disorders	76	(0.55)	51	(0.25)
	Mood disorders	58	(0.42)	55	(0.27)
	Neurotic, stress-related, and somatoform disorders	176	(1.28)	134	(0.67)
	Other Psychiatric disorder	355	(2.59)	198	(0.98)
	No psychiatric disorder	13,060	(95.15)	19,709	(97.83)
	**Highest obtained education**				
	Primary school^c^	5853	(42.64)	5244	(26.03)
	High school or vocational education	6364	(46.37)	10,356	(51.40)
	Short- or medium-cycle higher education	1119	(8.15)	3080	(15.29)
	Long-cycle higher education or PhD	389	(2.83)	1467	(7.28)
	**Working status**				
	Unemployed or otherwise outside the labor marked	2028	(14.78)	1657	(8.22)
	Student in education	166	(1.21)	264	(1.31)
	Blue collar worker	8000	(58.29)	9673	(48.01)
	Self-employed	641	(4.67)	1437	(7.13)
	Clerical worker or Leading wage-earner	2890	(21.06)	7116	(35.32)
	**Income**				
	Lowest quintile	4020	(29.29)	4014	(19.92)
	Second quintile	3064	(22.32)	4016	(19.93)
	Third quintile	2673	(19.48)	4028	(19.99)
	Fourth quintile	2186	(15.93)	4037	(20.04)
	Highest quintile	1782	(12.98)	4052	(20.11)

^a^The polygenic risk score of ADHD divided into fifty groups 1 to 50, here only showing counts for selected groups

^b^None of the controls had a mother with ADHD and 9 controls had a father with ADHD. Among the individuals with ADHD, 15 had a mother with ADHD and 49 had a father with ADHD. Definition of ADHD among the parents: ICD-10 diagnosis: F90 or ICD-8 diagnosis: 308.01

^c^This category includes individuals with missing information on parental education

**Fig. 2:**
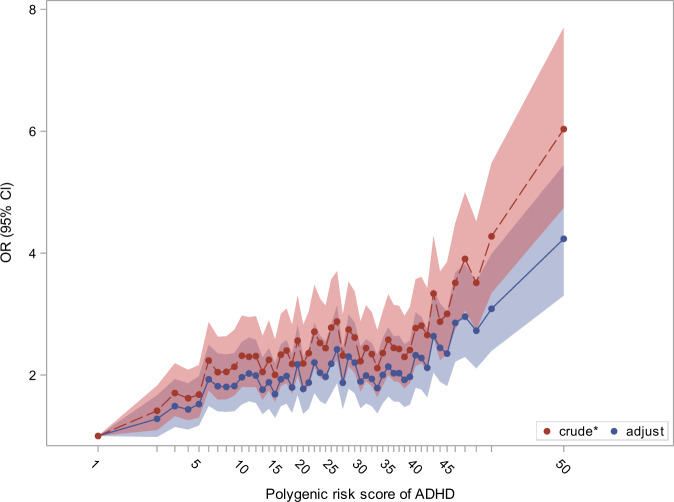
The crude and adjusted effect of the polygenic risk score for ADHD on the risk of ADHD. *crude adjustment: the estimates are only adjusted for sex and year of birth. The polygenic risk score for ADHD was divided into 50 groups each representing 2% of the distribution of the PRS for ADHD among the randomly drawn population controls.

Column 1 in Table 2 lists the crude odds ratios for ADHD (adjusted for sex and year of birth), related to the PRS for ADHD and the risk factors representing the psychosocial environment. Both the PRS for ADHD and the psychosocial factors were strongly associated with the risk of developing ADHD. Specifically, individuals with a PRS among the 2% highest values had a six fold increased risk of ADHD (6.03 (95% CI, 4.74–7.70)) compared to those with a PRS among the lowest 2%. The risk factors pertaining to the psychosocial environment (parental history of mental disorder, low parental education level, parental unemployment, and low parental income) were all associated with increased risk of ADHD at the statistically significant level. Column 2 lists odds ratios for ADHD for the risk factors representing the psychosocial environment after adjustment for the PRS, sex, and year of birth. This adjustment let to very subtle attenuations of the associations. Column 3 contains results from the mutually adjusted model. This led to modest attenuation of the association between the PRS and the risk for ADHD. For instance, the odds ratio associated with highest versus lowest 2% genetic liability decreased from 6.03 (95% CI, 4.74–7.70) to 4.23 (95% CI, 3.30–5.45). The crude and adjusted associations between the PRS and the risk of ADHD are shown in Fig. 2. The mutually adjusted odds ratios pertaining to the psychosocial environment should be interpreted with caution, as these factors are unlikely to be independent (e.g. labor market affiliation and income).

**Table 2:** Main effect of the polygenic risk score (PRS) and psychosocial risk factors on the risk of ADHD.

		Crude^a^		Prs adjusted^b^		Adjusted^c^	
		OR	(95% CL)	OR	(95% CL)	OR	(95% CL)
	**Sex**						
	Male	2.69	(2.57;2.82)	2.71	(2.58;2.84)	2.75	(2.61;2.89)
	Female	1.00	(ref)	1.00	(ref)	1.00	(ref)
	**Polygenic risk score**^**d**^						
	50	6.03	(4.74;7.70)	–	–	4.23	(3.30;5.45)
	40	2.77	(2.15;3.57)	–	–	2.33	(1.79;3.03)
	30	2.23	(1.73;2.88)	–	–	1.89	(1.46;2.47)
	20	2.19	(1.70;2.83)	–	–	1.77	(1.37;2.31)
	10	2.32	(1.81;2.98)	–	–	1.96	(1.52;2.54)
	1	1.00	(ref)	–	–	1.00	(ref)
Mother	**History of mental disorder**						
	Schizophrenia and related disorders	1.99	(1.37;2.92)	1.97	(1.35;2.91)	1.23	(0.83;1.84)
	Mood disorders	1.67	(1.24;2.25)	1.66	(1.23;2.24)	1.40	(1.03;1.90)
	Neurotic, stress-related, and somatoform disorders	2.29	(1.90;2.76)	2.28	(1.90;2.75)	1.70	(1.41;2.07)
	Other Psychiatric disorder	2.31	(1.90;2.81)	2.23	(1.83;2.72)	1.51	(1.23;1.86)
	No psychiatric disorder	1.00	(ref)	1.00	(ref)	1.00	(ref)
	**Highest obtained education**						
	Primary school^e^	5.90	(5.04;6.94)	5.63	(4.80;6.63)	2.24	(1.87;2.70)
	High school or vocational education	2.84	(2.43;3.33)	2.75	(2.35;3.23)	1.54	(1.29;1.85)
	Short- or medium-cycle higher education	1.85	(1.57;2.18)	1.83	(1.56;2.17)	1.39	(1.17;1.66)
	Long-cycle higher education or PhD	1.00	(ref)	1.00	(ref)	1.00	(ref)
	**Working status**						
	Unemployed or otherwise outside the labor marked	2.76	(2.59;2.94)	2.69	(2.52;2.86)	1.45	(1.34;1.57)
	Student in education	2.29	(2.00;2.62)	2.27	(1.98;2.60)	1.43	(1.23;1.67)
	Blue collar worker	1.98	(1.87;2.09)	1.94	(1.83;2.05)	1.24	(1.16;1.33)
	Self-employed	1.24	(1.02;1.50)	1.22	(1.00;1.48)	1.00	(0.81;1.22)
	Clerical worker or Leading wage-earner	1.00	(ref)	1.00	(ref)	1.00	(ref)
	**Income**						
	Lowest quintile	2.67	(2.47;2.88)	2.61	(2.42;2.82)	1.20	(1.09;1.31)
	Second quintile	2.12	(1.96;2.28)	2.07	(1.91;2.23)	1.12	(1.02;1.22)
	Third quintile	1.79	(1.66;1.94)	1.76	(1.63;1.90)	1.12	(1.03;1.22)
	Fourth quintile	1.47	(1.35;1.59)	1.45	(1.34;1.57)	1.13	(1.04;1.23)
	Highest quintile	1.00	(ref)	1.00	(ref)	1.00	(ref)
Father	**History of mental disorder**						
	Schizophrenia and related disorders	2.22	(1.54;3.23)	2.22	(1.54;3.24)	1.51	(1.02;2.25)
	Mood disorders	1.78	(1.21;2.62)	1.78	(1.20;2.62)	1.46	(0.96;2.21)
	Neurotic, stress-related, and somatoform disorders	1.88	(1.49;2.38)	1.86	(1.47;2.35)	1.27	(0.99;1.62)
	Other Psychiatric disorder	2.90	(2.42;3.48)	2.84	(2.37;3.42)	1.71	(1.41;2.08)
	No psychiatric disorder	1.00	(ref)	1.00	(ref)	1.00	(ref)
	**Highest obtained education**						
	Primary school^e^	4.59	(4.07;5.19)	4.35	(3.86;4.93)	1.94	(1.68;2.24)
	High school or vocational education	2.43	(2.16;2.74)	2.34	(2.08;2.65)	1.40	(1.22;1.61)
	Short- or medium-cycle higher education	1.37	(1.20;1.56)	1.34	(1.17;1.54)	1.10	(0.95;1.26)
	Long-cycle higher education or PhD	1.00	(ref)	1.00	(ref)	1.00	(ref)
	**Working status**						
	Unemployed or otherwise outside the labor marked	3.18	(2.93;3.45)	3.07	(2.83;3.33)	1.34	(1.21;1.48)
	Student in education	1.51	(1.23;1.85)	1.52	(1.24;1.87)	0.94	(0.75;1.18)
	Blue collar worker	1.99	(1.88;2.10)	1.95	(1.84;2.06)	1.20	(1.12;1.28)
	Self-employed	1.10	(0.99;1.22)	1.10	(0.99;1.22)	0.80	(0.71;0.89)
	Clerical worker or Leading wage-earner	1.00	(ref)	1.00	(ref)	1.00	(ref)
	**Income**						
	Lowest quintile	2.51	(2.33;2.70)	2.44	(2.27;2.63)	1.21	(1.10;1.32)
	Second quintile	1.92	(1.78;2.07)	1.89	(1.75;2.05)	1.14	(1.05;1.24)
	Third quintile	1.61	(1.49;1.74)	1.59	(1.47;1.72)	1.11	(1.02;1.20)
	Fourth quintile	1.26	(1.16;1.36)	1.24	(1.15;1.35)	0.98	(0.90;1.06)
	Highest quintile	1.00	(ref)	1.00	(ref)	1.00	(ref)

^a^Adjustment for sex and year of birth

^b^Adjustment for sex, year of birth and the polygenic risk score for ADHD

^c^All estimates are mutually adjusted and adjusted for year of birth

^d^The polygenic risk score of ADHD divided into fifty groups 1 to 50, here only showing estimates for selected groups (see Fig. 2 for more details)

^e^This category includes individuals with missing information on parental education

The population marginal means of the PRS for ADHD adjusted for sex and year of birth are displayed in Figure S1, and show that the polygenic liability was higher among individuals with ADHD than controls across all of the risk factors pertaining to the psychosocial environment. These differences were less apparent for those having parents with mental disorders, but more pronounced across the other environmental factors with a weak tendency to a lower liability in subjects whose parents had longer educational attainment or higher income.

**Fig. 3:**
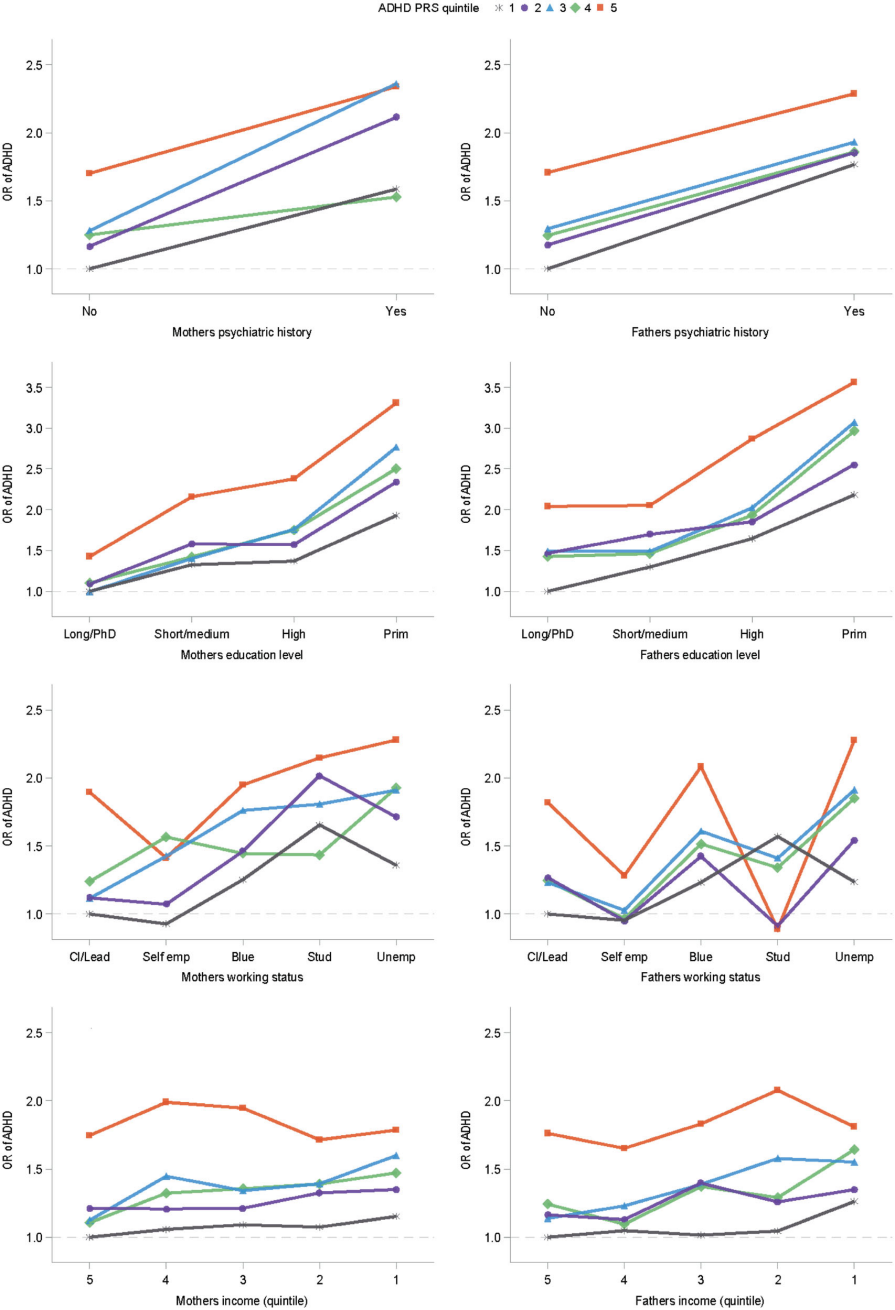
Polygenic Risk Score and Parental Psychosocial Environment. Odds ratio for ADHD across levels of parental risk factors and polygenic risk score quartiles.

The combined effect of the PRS for ADHD in quintiles and each of the psychosocial risk factors (mutually adjusted for) is shown in Fig. 3.

These plots are predominantly indicative of independence between the effects of the PRS for ADHD and the psychosocial risk factors upon the risk of ADHD (the *p*-values for interaction range from 0.06 to 0.48).

The results listed in Table S1, Table S2, Figs. S2, S3, and S4 in the supplementary material are analog to those in Tables 1 and 2, Figs. 1, 2 and 3; however, without excluding the ancestral principal components outliers. The results in these two sets of analyses are practically identical. As a representative example, the odds ratio for ADHD associated with the highest versus the lowest 2% genetic liability was 6.01 (95%CI, 4.77–7.60) prior to excluding the ancestral principal components outliers, and 6.03 (95%CI, 4.74–7.70) after excluding these outliers.

## Discussion

In this population-based study, we found the expected main effects of both polygenic liability for ADHD and of risk factors related to the psychosocial environment (maternal/paternal education, maternal/paternal work status, maternal/paternal income, and maternal/paternal history of mental disorder) upon the risk of ADHD. Furthermore, the associations with the PRS for ADHD and paternal education, paternal income, and maternal work status were largely independent with little indication of any gene–environment interaction.

That the PRS for ADHD is strongly associated with the development of ADHD in this sample is consistent with a substantial body of evidence suggesting that ADHD is among the most heritable conditions among the mental disorders^11–13^. Also, it is in line with the recent GWAS, which identified the first genome-wide significant loci for ADHD^15^. Notably, the association between the PRS for ADHD and the risk for ADHD reported here remained almost unchanged when adjusting for the psychosocial risk factors, which included the maternal and paternal history of mental disorder. Thus, the PRS effects were not explained by parental psychopathology or socioeconomic factors, and furthermore, the impacts of the psychosocial factors were not mediated the genetic liability for ADHD. This suggests that manifest mental disorder in the parents is not a requirement for transmission of genetic risk for ADHD^12,13^.

Psychosocial adversity operationalized in a vast variety of ways has been associated with increased risk of ADHD in a large number of studies^17–21^. The present study corroborates these findings using a relatively broad definition of psychosocial adversity, which likely taps into a “background” environment where some of the more specific insults that have been associated with subsequent development of ADHD—such as early severe deprivation^55^, maltreatment^56^, and inconsistent parenting^24^—are also more likely to occur.

In this study, we found no support for interaction between the polygenic liability for ADHD and the psychosocial environment upon the risk of ADHD. This is in contrast with results from prior studies that have investigated the role of gene–environment interaction in the etiology of ADHD^15,25–29^. However, these prior studies have (i) tended to be based on small case-healthy control samples, (ii) used self-reported historical data on psychosocial environment, and (iii) focused on individual candidate genes (predominantly in the dopaminergic and serotonergic system) that were not replicated in the recent GWAS of ADHD^15,25–29^. For these reasons, the results of the present study are most likely more internally valid than those from the prior studies on this topic.

The results of this study raise the question as to which specific genetic pathways are responsible for the observed dose-response association between the PRS for ADHD and development of ADHD. Based on the results from the recent GWAS of ADHD^15^, genes involved in synapse formation (*FOXP2*)^57,58^, neuroplasticity (*SORCS3*)^59,60^, and dopaminergic homeostasis (*DUSP6*)^61,62^ may be playing an important role. Determining whether this is indeed the case, will require larger GWAS of ADHD to allow for sufficient estimation of PRSs for specific genetic pathways.

There are limitations to this study, which must be taken into account by the reader. First, due to the biobank- and register-based nature of this study, we were only able to include individuals with ADHD who received a diagnosis of ADHD during inpatient or outpatient treatment at a psychiatric hospital in Denmark^32^. Hence, children who were diagnosed with ADHD outside psychiatric hospital settings, e.g. by private practicing psychiatrists or by pediatricians, do not appear as “cases” in this study (false negatives)—as these practitioners do not report diagnoses to the Danish Psychiatric Central Research Register, which provided the diagnostic data for this study. Therefore, our results might not generalize to patients with less severe ADHD than those diagnosed at psychiatric hospitals. On the other hand, an advantage of using register data is that the validity of the ADHD diagnoses in the register has been evaluated and found to be appropriate for research purposes^63,64^. Secondly, gene–environment correlation may threaten the internal validity of the results^65^. Specifically, the parents of the cohort members may create a family environment that is correlated with the cohort members’ genotype—an effect of so-called genetic nurture, in which environmental influences are misidentified as genetic^66^ In order to counteract this effect, we have adjusted the analyses involving the PRS for ADHD for the parents’ history of diagnosed mental disorder. However, since this adjustment only takes relatively severe psychopathology into account, which has led to treatment at psychiatric hospitals, this does not rule out the possibility that passive, or evocative^67^, gene–environment correlation may have led to an overestimation of the effect of gene–environment interactions on the risk of ADHD due to confounding^65^. Third and finally, it is broadly recognized that PRSs are not yet very informative at the level of the individual and therefore not clinically useful in psychiatry^68,69^. However, this may change with the results of future GWAS that will likely to explain a larger fraction of the polygenic liability for ADHD and other mental disorders.

In conclusion, based on a study of 13,725 individuals with ADHD and 20,147 population controls, we report strong and independent main effects of genetic liability and psychosocial adversity upon the risk of ADHD—in the expected direction. In contrast, we found no significant interactions between the polygenic liability and the psychosocial environment upon the risk of ADHD, and the genetic liability in individuals with ADHD was increased across all psychosocial factors. Future studies should address if specific genetic pathways and environmental factors are responsible for these results. This line of research may lead to identification of targets for both treatment and preventive measures.

## Supplementary information

Supplementary Material
