# Supplementary material for: Polygenic risk score, psychosocial environment and the risk of attention-deficit/hyperactivity disorder
Source: Transl Psychiatry. 2020 Oct 2;10:335. doi: 10.1038/s41398-020-01019-6 (PMC7532146; doi:10.1038/s41398-020-01019-6)
Supplement: Supplementary file 1 — Supplementary Material [file 41398_2020_1019_MOESM1_ESM.docx]

**Supplemental material**

**Figure S1. The polygenic liability for ADHD among individuals with ADHD (red) and randomly drawn population controls (blue) across the risk factors pertaining to the psychosocial environment.**


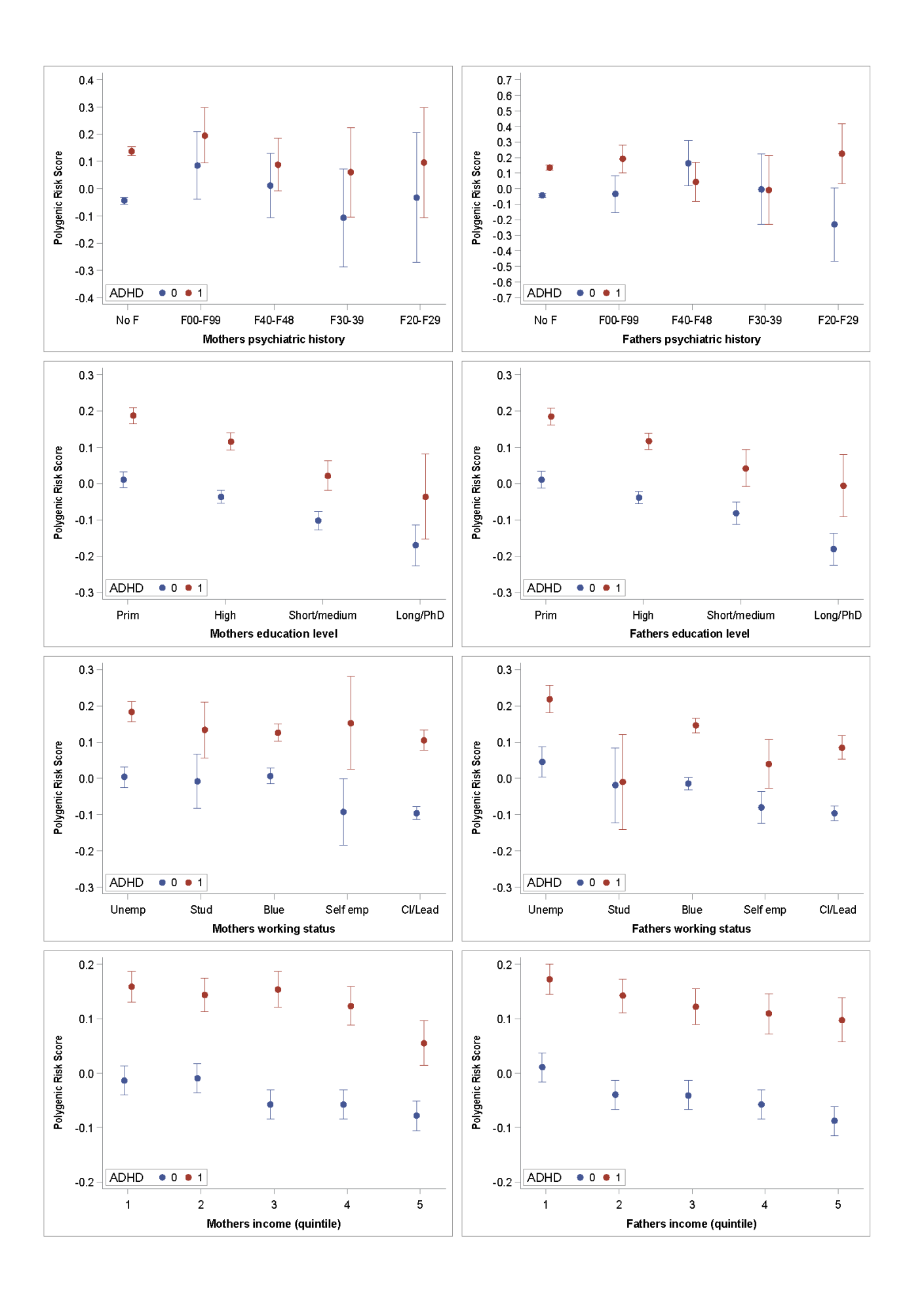


**Table S1. Distribution of polygenic risk score, parental history of mental disorders and socioeconomic factors for the ADHD cases and the randomly drawn population controls, when not excluding ancestral principal components outliers.**

|  |  | ADHD cases | |  | Controls | |
| --- | --- | --- | --- | --- | --- | --- |
|  |  | N | (%) |  | N | (%) |
|  | **Sex** |  |  |  |  |  |
|  | Male | 10,942 | (73.71) |  | 11,089 | (50.69) |
|  | Female | 3,903 | (26.29) |  | 10,788 | (49.31) |
|  |  |  |  |  |  |  |
|  | **Polygenic risk score^a^** |  |  |  |  |  |
|  | 50 | 560 | (3.77) |  | 369 | (1.69) |
|  | 40 | 285 | (1.92) |  | 418 | (1.91) |
|  | 30 | 260 | (1.75) |  | 448 | (2.05) |
|  | 20 | 275 | (1.85) |  | 463 | (2.12) |
|  | 10 | 302 | (2.03) |  | 448 | (2.05) |
|  | 1 | 168 | (1.13) |  | 470 | (2.15) |
|  |  |  |  |  |  |  |
| Mother | **History of mental disorder** |  |  |  |  |  |
|  | Schizophrenia and related disorders | 78 | (0.53) |  | 51 | (0.23) |
|  | Mood disorders | 113 | (0.76) |  | 97 | (0.44) |
|  | Neurotic, stress-related, and somatoform disorders | 338 | (2.28) |  | 229 | (1.05) |
|  | Other Psychiatric disorder | 304 | (2.05) |  | 195 | (0.89) |
|  | No psychiatric disorder | 14,012 | (94.39) |  | 21,305 | (97.39) |
|  | **Highest obtained education** |  |  |  |  |  |
|  | Primary school^b^ | 6,969 | (46.95) |  | 6,514 | (29.78) |
|  | High school or vocational education | 5,789 | (39.00) |  | 9,569 | (43.74) |
|  | Short- or medium-cycle higher education | 1,854 | (12.49) |  | 4,820 | (22.03) |
|  | Long-cycle higher education or PhD | 233 | (1.57) |  | 974 | (4.45) |
|  | **Working status** |  |  |  |  |  |
|  | Unemployed or otherwise outside the labor marked | 4,174 | (28.12) |  | 3,817 | (17.45) |
|  | Student in education | 523 | (3.52) |  | 553 | (2.53) |
|  | Blue collar worker | 5,874 | (39.57) |  | 7,024 | (32.11) |
|  | Self employed | 192 | (1.29) |  | 384 | (1.76) |
|  | Clerical worker or Leading wage-earner | 4,082 | (27.50) |  | 10,099 | (46.16) |
|  | **Income** |  |  |  |  |  |
|  | Lowest quintile | 4,044 | (27.24) |  | 4,362 | (19.94) |
|  | Second quintile | 3,384 | (22.80) |  | 4,368 | (19.97) |
|  | Third quintile | 2,973 | (20.03) |  | 4,375 | (20.00) |
|  | Fourth quintile | 2,558 | (17.23) |  | 4,382 | (20.03) |
|  | Highest quintile | 1,886 | (12.70) |  | 4,390 | (20.07) |
|  |  |  |  |  |  |  |
| Father | **History of mental disorder** |  |  |  |  |  |
|  | Schizophrenia and related disorders | 81 | (0.55) |  | 53 | (0.24) |
|  | Mood disorders | 65 | (0.44) |  | 60 | (0.27) |
|  | Neurotic, stress-related, and somatoform disorders | 195 | (1.31) |  | 149 | (0.68) |
|  | Other Psychiatric disorder | 377 | (2.54) |  | 217 | (0.99) |
|  | No psychiatric disorder | 14,127 | (95.16) |  | 21,398 | (97.81) |
|  | **Highest obtained education** |  |  |  |  |  |
|  | Primary school or missing | 6,372 | (42.92) |  | 5,720 | (26.15) |
|  | High school or vocational education | 6,835 | (46.04) |  | 11,240 | (51.38) |
|  | Short- or medium-cycle higher education | 1,211 | (8.16) |  | 3,327 | (15.21) |
|  | Long-cycle higher education or PhD | 427 | (2.88) |  | 1,590 | (7.27) |
|  | **Working status** |  |  |  |  |  |
|  | Unemployed or otherwise outside the labor marked | 2,211 | (14.89) |  | 1,810 | (8.27) |
|  | Student in education | 183 | (1.23) |  | 290 | (1.33) |
|  | Blue collar worker | 8,645 | (58.24) |  | 10,495 | (47.97) |
|  | Self employed | 698 | (4.70) |  | 1,592 | (7.28) |
|  | Clerical worker or Leading wage-earner | 3,108 | (20.94) |  | 7,690 | (35.15) |
|  | **Income** |  |  |  |  |  |
|  | Lowest quintile | 4,375 | (29.47) |  | 4,357 | (19.92) |
|  | Second quintile | 3,336 | (22.47) |  | 4,360 | (19.93) |
|  | Third quintile | 2,870 | (19.33) |  | 4,376 | (20.00) |
|  | Fourth quintile | 2,356 | (15.87) |  | 4,383 | (20.03) |
|  | Highest quintile | 1,908 | (12.85) |  | 4,401 | (20.12) |
|  |  |  |  |  |  |  |

^a^ The polygenic risk score of ADHD divided into fifty groups 1 to 50, here only showing counts for selected groups.

^b^ This category includes individuals with missing information on parental education.

### Table S2. Main effect of the polygenic risk score and psychosocial risk factors on the risk of ADHD, when not excluding ancestral principal components outliers.

|  |  | Crude^a^ | |  | | Prs adjusted^b^ | | |  | Adjusted^c^ | |
| --- | --- | --- | --- | --- | --- | --- | --- | --- | --- | --- | --- |
|  |  | OR | (95%CL) |  | | OR | (95%CL) | |  | OR | (95%CL) |
|  | **Sex** |  |  |  | |  |  | |  |  |  |
|  | Male | 2.68 | (2.56;2.80) |  | | 2.70 | (2.58;2.83) | |  | 2.73 | (2.60;2.87) |
|  | Female | 1.00 | (ref) |  | | 1.00 | (ref) | |  | 1.00 | (ref) |
|  |  |  |  |  | |  |  | |  |  |  |
|  | **Polygenic risk score^d^** |  |  |  | |  |  | |  |  |  |
|  | 50 | 6.01 | (4.77;7.60) |  | | - | - | |  | 4.15 | (3.27;5.28) |
|  | 40 | 2.70 | (2.12;3.45) |  | | - | - | |  | 2.22 | (1.73;2.86) |
|  | 30 | 2.17 | (1.70;2.78) |  | | - | - | |  | 1.84 | (1.43;2.37) |
|  | 20 | 2.22 | (1.74;2.83) |  | | - | - | |  | 1.80 | (1.40;2.31) |
|  | 10 | 2.25 | (1.78;2.86) |  | | - | - | |  | 1.91 | (1.49;2.44) |
|  | 1 | 1.00 | (ref) |  | | - | - | |  | 1.00 | (ref) |
|  |  |  |  |  | |  |  | |  |  |  |
| Mother | **History of mental disorder** |  |  |  | |  |  | |  |  |  |
|  | Schizophrenia and related disorders | 2.26 | (1.57;3.29) |  | | 2.24 | (1.55;3.26) | |  | 1.43 | (0.97;2.12) |
|  | Mood disorders | 1.64 | (1.24;2.18) |  | | 1.63 | (1.23;2.17) | |  | 1.38 | (1.03;1.85) |
|  | Neurotic, stress-related, and somatoform disorders | 2.21 | (1.86;2.64) |  | | 2.20 | (1.84;2.63) | |  | 1.64 | (1.37;1.98) |
|  | Other Psychiatric disorder | 2.40 | (1.99;2.90) |  | | 2.32 | (1.92;2.81) | |  | 1.58 | (1.30;1.93) |
|  | No psychiatric disorder | 1.00 | (ref) |  | | 1.00 | (ref) | |  | 1.00 | (ref) |
|  | **Highest obtained education** |  |  |  | |  |  | |  |  |  |
|  | Primary school^e^ | 5.66 | (4.87;6.60) |  | | 5.39 | (4.64;6.29) | |  | 2.19 | (1.84;2.62) |
|  | High school or vocational education | 2.72 | (2.34;3.17) |  | | 2.64 | (2.27;3.08) | |  | 1.51 | (1.28;1.79) |
|  | Short- or medium-cycle higher education | 1.74 | (1.49;2.04) |  | | 1.73 | (1.48;2.03) | |  | 1.33 | (1.12;1.57) |
|  | Long-cycle higher education or PhD | 1.00 | (ref) |  | | 1.00 | (ref) | |  | 1.00 | (ref) |
|  | **Working status** |  |  |  | |  |  | |  |  |  |
|  | Unemployed or otherwise outside the labor marked | 2.67 | (2.48;2.87) |  | | 2.68 | (2.53;2.85) | |  | 1.43 | (1.33;1.55) |
|  | Student in education | 2.26 | (1.98;2.57) |  | | 2.24 | (1.96;2.55) | |  | 1.41 | (1.22;1.63) |
|  | Blue collar worker | 1.97 | (1.87;2.08) |  | | 1.93 | (1.83;2.04) | |  | 1.23 | (1.16;1.32) |
|  | Self employed | 1.20 | (1.00;1.44) |  | | 1.18 | (0.98;1.42) | |  | 0.97 | (0.80;1.17) |
|  | Clerical worker or Leading wage-earner | 1.00 | (ref) |  | | 1.00 | (ref) | |  | 1.00 | (ref) |
|  | **Income** |  |  |  | |  |  | |  |  |  |
|  | Lowest quintile | 2.67 | (2.48;2.87) |  | | 2.60 | (2.42;2.80) | |  | 1.19 | (1.09;1.30) |
|  | Second quintile | 2.12 | (1.97;2.28) |  | | 2.07 | (1.92;2.23) | |  | 1.11 | (1.02;1.21) |
|  | Third quintile | 1.80 | (1.67;1.94) |  | | 1.77 | (1.64;1.91) | |  | 1.12 | (1.03;1.21) |
|  | Fourth quintile | 1.47 | (1.36;1.59) |  | | 1.46 | (1.35;1.57) | |  | 1.13 | (1.04;1.22) |
|  | Highest quintile | 1.00 | (ref) |  | | 1.00 | (ref) | |  | 1.00 | (ref) |
|  |  |  |  |  | |  |  | |  |  |  |
| Father | **History of mental disorder** |  |  |  | |  |  | |  |  |  |
|  | Schizophrenia and related disorders | 2.27 | (1.59;3.28) |  | | 2.25 | (1.57;3.25) | |  | 1.52 | (1.04;2.25) |
|  | Mood disorders | 1.80 | (1.24;2.60) |  | | 1.79 | (1.24;2.60) | |  | 1.49 | (1.00;2.21) |
|  | Neurotic, stress-related, and somatoform disorders | 1.85 | (1.48;2.31) |  | | 1.82 | (1.45;2.28) | |  | 1.24 | (0.99;1.57) |
|  | Other Psychiatric disorder | 2.83 | (2.37;3.37) |  | | 2.77 | (2.33;3.32) | |  | 1.68 | (1.40;2.02) |
|  | No psychiatric disorder | 1.00 | (ref) |  | | 1.00 | (ref) | |  | 1.00 | (ref) |
|  | **Highest obtained education** |  |  |  | |  |  | |  |  |  |
|  | Primary school or missing | 4.53 | (4.03;5.09) |  | | 4.29 | (3.82;4.83) | |  | 1.90 | (1.65;2.18) |
|  | High school or vocational education | 2.37 | (2.12;2.66) |  | | 2.29 | (2.04;2.57) | |  | 1.36 | (1.20;1.56) |
|  | Short- or medium-cycle higher education | 1.36 | (1.19;1.54) |  | | 1.33 | (1.17;1.51) | |  | 1.09 | (0.95;1.25) |
|  | Long-cycle higher education or PhD | 1.00 | (ref) |  | | 1.00 | (ref) | |  | 1.00 | (ref) |
|  | **Working status** |  | |  |  | | |  |  |  |  |
|  | Unemployed or otherwise outside the labor marked | 3.16 | (2.92;3.42) |  | | 3.04 | (2.81;3.29) | |  | 1.32 | (1.20;1.46) |
|  | Student in education | 1.52 | (1.25;1.85) |  | | 1.53 | (1.25;1.86) | |  | 0.93 | (0.75;1.14) |
|  | Blue collar worker | 1.98 | (1.88;2.09) |  | | 1.95 | (1.85;2.05) | |  | 1.20 | (1.13;1.28) |
|  | Self employed | 1.08 | (0.98;1.20) |  | | 1.08 | (0.97;1.20) | |  | 0.79 | (0.71;0.88) |
|  | Clerical worker or Leading wage-earner | 1.00 | (ref) |  | | 1.00 | (ref) | |  | 1.00 | (ref) |
|  | **Income** |  |  |  | |  |  | |  |  |  |
|  | Lowest quintile | 2.56 | (2.38;2.75) |  | | 2.48 | (2.31;2.67) | |  | 1.24 | (1.14;1.35) |
|  | Second quintile | 1.97 | (1.83;2.12) |  | | 1.94 | (1.81;2.09) | |  | 1.18 | (1.09;1.28) |
|  | Third quintile | 1.62 | (1.50;1.75) |  | | 1.60 | (1.49;1.73) | |  | 1.12 | (1.04;1.22) |
|  | Fourth quintile | 1.27 | (1.18;1.37) |  | | 1.26 | (1.17;1.36) | |  | 0.99 | (0.91;1.07) |
|  | Highest quintile | 1.00 | (ref) |  | | 1.00 | (ref) | |  | 1.00 | (ref) |
|  |  |  |  |  | |  |  | |  |  |  |

^a^ Adjustment for sex and year of birth.

^b^ Adjustment for sex, year of birth and the polygenic risk score for ADHD.

^c^ All estimates are mutually adjusted and adjusted for year of birth.

^d^ The polygenic risk score of ADHD divided into fifty groups 1 to 50, here only showing estimates for selected groups (see figure S3 for more details).

^e^ This category includes individuals with missing information on parental education.

### Figure S2. The distribution of the polygenic risk score (PRS) for ADHD among cases (red) and randomly drawn population controls (blue), when not excluding ancestral principal components outliers.

### Figure S3. The crude and adjusted effect of the polygenic risk score for ADHD on the risk of ADHD, when not excluding ancestral principal components outliers.

*crude adjustment: the estimates are only adjusted for sex and year of birth.

The polygenic risk score for ADHD was divided into 50 groups each representing 2% of the distribution of the PRS for ADHD among the randomly drawn population controls.

### Figure S4. The combined effect of the polygenic risk score for ADHD and psychosocial risk factors upon the risk of ADHD, when not excluding ancestral principal components outliers.

**
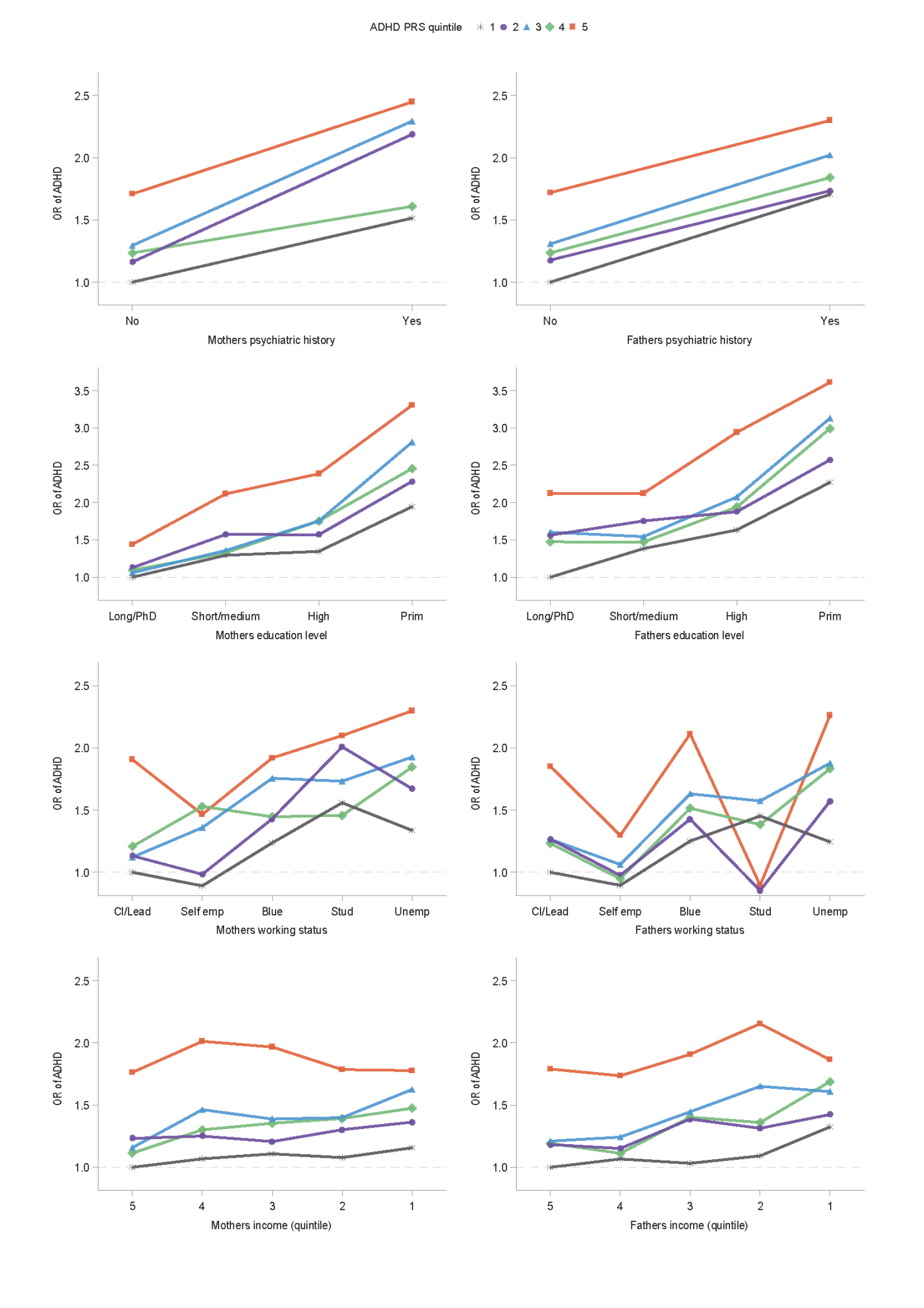
**
